# Supplementary material for: Transcriptome Analysis of the Responses of Rice Leaves to Chilling and Subsequent Recovery
Source: Int J Mol Sci. 2022 Sep 15;23(18):10739. doi: 10.3390/ijms231810739 (PMC9502032; doi:10.3390/ijms231810739)

Table S1 The chlorophyll a, b and a+b contents in Dular under three different temperature treatments

Note:

Treatment	chlorophyll a	chlorophyll b	chlorophyll a+b	carotenoid
CT	0.548 ± 0.023 a	0.279 ± 0.014 a	0.828 ± 0.031 a	0.072 ± 0.006 a
TR	0.130 ± 0.002 c	0.145 ± 0.001 b	0.275 ± 0.003 c	0.006 ± 0.001 c
RC	0.207 ± 0.011 b	0.163 ± 0.006 b	0.369 ± 0.012 b	0.026 ± 0.001 b

Unit: $mg \cdot g^{-1} \cdot FW$

CT: Normal-temperature treatment.

TR: Low-temperature treatment.

RC: Recovery to normal-temperature treatment

Columns with different letters are significantly different (LSD test, $p < 0.05$).

Table S2 The number of genes identification in Dular under three different temperature treatments

Treatment	Total DEGs	Only in CT, TR or RC	Both in CT and TR	Both in TR and RC	Both in CT and RC	Synchronously expressed across CT, TR and RC
CT	20096	1065	839	733	444	17748
TR	20177	857				
RC	19209	284				

Note:

CT: Normal-temperature treatment.

TR: Low-temperature treatment.

RC: Recovery to normal-temperature treatment

Table S3 The number of different expression genes in Dular under three different temperature treatments

Treatment	Total DEGs	Up	Down	Only in TR/CT or RC/TR	Both in TR/CT and RC/TR
TR/CT	9143	5524	3619	5641	3502
RC/TR	6091	2789	3302	2589	

Note:

CT: Normal-temperature treatment.

TR: Low-temperature treatment.

RC: Recovery to normal-temperature treatment

Table S4 The differentially expressed genes under chilling and /or subsequent recovery

Gene ID	Describe	Subcellular location	Pathway ID	Genes expression			Log2 (Fold change)	
				CT	TR	RC	TR/CT	RC/TR
alpha-Linolenic acid metabolism								
BGIOSGA018751	Acyl-coenzyme A oxidase 4	Peroxisome	map00592	55.650	13.217	23.533	-2.074 (-)	0.832 (*)
BGIOSGA037332	Alpha-dioxygenase 1	monolayer-surrounded lipid storage body	map00592	10.453	19.190	62.567	0.876 (*)	1.705 (+)
BGIOSGA021674	12-oxophytodienoate reductase 5	Peroxisome	map00592	7.320	5.593	0.663	-0.388 (*)	-3.077 (-)
BGIOSGA006958	Lipoxygenase 2.3	chloroplastic	map00592	28.737	5.963	37.103	-2.269 (-)	2.637 (+)
BGIOSGA001629	12-oxophytodienoate reductase 10	Peroxisome	map00592	129.917	4.937	11.167	-4.718 (-)	1.178 (+)
BGIOSGA013421	Phospholipase A2 homolog 3	Endoplasmic reticulum	map00592	12.750	42.657	8.210	1.742 (+)	-2.377 (-)
BGIOSGA007800	Allene oxide synthase 4	Chloroplast	map00592	0.013	6.507	0.277	8.967 (+)	-4.554 (-)
BGIOSGA007799	Allene oxide synthase 3	Chloroplast	map00592	0.047	1.150	3.540	4.613 (+)	1.622 (+)
BGIOSGA038638	Jasmonate O-methyltransferase	Cytoplasm	map00592	1.697	13.903	4.897	3.034 (+)	-1.505 (-)
BGIOSGA000776	Triacylglycerol lipase SDP1	Peroxisome	map00592	7.977	2.150	5.477	-1.892 (-)	1.349 (+)
BGIOSGA034308	Alcohol dehydrogenase 2	Unkown	map00592	17.683	17.060	2.313	-0.052 (*)	-2.883 (-)
BGIOSGA021672	Putative 12-oxophytodienoate reductase 4	Unkown	map00592	17.313	15.930	2.943	-0.120 (*)	-2.436 (-)
Carbon fixation in photosynthetic organisms								
BGIOSGA034421	Fructose-bisphosphate aldolase	chloroplastic	map00710	1140.460	3280.660	7827.193	1.524 (+)	1.255 (+)
BGIOSGA006800	Malate dehydrogenase	chloroplastic	map00710	10.913	25.110	79.393	1.202 (+)	1.661 (+)
BGIOSGA016636	Glyceraldehyde-3-phosphate dehydrogenase 2	cytosolic	map00710	74.037	438.067	173.340	2.565 (+)	-1.338 (-)

BGIOSGA004865	Fructose-1,6-bisphosphatase	cytosolic	map00710	92.643	213.637	674.463	1.205 (+)	1.659 (+)
BGIOSGA016887	Malate dehydrogenase	cytoplasmic	map00710	2.303	6.410	2.433	1.477 (+)	-1.397 (+)
BGIOSGA004108	Malate dehydrogenase	mitochondrial	map00710	70.433	167.693	49.720	1.251 (+)	-1.754 (+)
BGIOSGA002229	NADP-dependent malic enzyme	chloroplastic	map00710	33.023	91.857	180.060	1.476 (+)	0.971 (*)
BGIOSGA010483	Pyruvate phosphate dikinase	Chloroplast	map00710	0.820	8.427	2.057	3.361 (+)	-2.034 (-)
BGIOSGA032182	Phosphoenolpyruvate carboxykinase	Cytoplasm	map00710	0.287	9.047	0.943	4.978 (+)	-3.262 (-)
BGIOSGA004733	Malate dehydrogenase	chloroplastic	map00710	2.590	7.257	1.347	1.486 (+)	-2.430 (-)
BGIOSGA037061	Fructose-bisphosphate aldolase	chloroplastic	map00710	3.607	4.617	101.920	0.356 (*)	4.464 (+)
BGIOSGA037062	Fructose-bisphosphate aldolase 2	chloroplastic	map00710	14.080	20.260	289.333	0.525 (*)	3.836 (+)
BGIOSGA023247	Fructose-bisphosphate aldolase 5	cytosolic	map00710	253.223	236.610	652.247	-0.098 (*)	1.463 (+)
BGIOSGA008865	Phosphoribulokinase	chloroplastic	map00710	883.963	636.560	2222.373	-0.474 (*)	1.804 (+)
BGIOSGA038154	Ribulose bisphosphate carboxylase small chain A	chloroplastic	map00710	6111.967	3809.640	11125.280	-0.682 (*)	1.546 (+)
Diterpenoid biosynthesis								
BGIOSGA017095	Terpene_synth_C domain-containing protein	Chloroplast	map00904	0.050	0.260	1.743	2.379 (+)	2.745 (+)
BGIOSGA002270	Gibberellin 3-beta-dioxygenase 2-2	Unkown	map00904	1.040	2.987	0.863	1.522 (+)	-1.791 (-)
BGIOSGA015502	Uncharacterized protein	Unkown	map00904	0.420	0.820	4.390	0.965 (*)	2.421 (+)
BGIOSGA008469	Ent-copalyl diphosphate synthase 2	Chloroplast	map00904	4.507	0.617	10.420	-2.869 (-)	4.078 (+)
BGIOSGA008468	Ent-isokaurene C2-hydroxylase	Membrane	map00904	23.980	4.070	15.043	-2.559 (-)	1.886 (+)
BGIOSGA014695	Gibberellin 2-beta-dioxygenase 8	Cytoplasm	map00904	2.017	8.027	2.507	1.993 (+)	-1.679 (-)
BGIOSGA013985	Gibberellin 20 oxidase 1	cytoplasm	map00904	8.977	21.627	7.933	1.269 (+)	-1.447 (-)
BGIOSGA017571	Fe_2OG dioxygenase domain-containing protein	Cytosol	map00904	0.733	2.557	5.830	1.803 (+)	1.189 (+)

BGIOSGA017729	Gibberellin 2-beta-dioxygenase	Cytoplasm	map00904	0.140	11.283	3.277	6.333 (+)	-1.784 (-)
BGIOSGA002149	Gibberellin 2-beta-dioxygenase 1	Cytoplasm	map00904	1.050	2.347	0.523	1.160 (+)	-2.166 (-)
BGIOSGA021012	Ent-kaurene oxidase-like 5	Membrane	map00904	0.557	0.843	8.717	0.598 (*)	3.370 (+)
BGIOSGA008466	Ent-cassadiene C11-alpha-hydroxylase 1	Membrane	map00904	0.333	0.420	1.263	0.335 (*)	1.588 (+)
BGIOSGA015981	9-beta-pimara-7,15-diene oxidase	Membrane	map00904	1.827	0.580	2.047	-1.655 (-)	1.819 (+)
BGIOSGA015502	Syn-copalyl diphosphate synthase	Chloroplast	map00904	0.420	0.820	4.390	0.965 (*)	2.421 (+)
BGIOSGA006215	Ent-cassadiene hydroxylase	Membrane	map00904	1.247	0.663	3.643	-0.911 (*)	2.458 (+)
BGIOSGA034012	KSL11_ORYSI(Stemod-13(17)-ene synthase	Unkown	map00904	1.430	1.233	8.953	-0.214 (*)	2.860 (+)
Glyoxylate and dicarboxylate metabolism								
BGIOSGA027522	aconitate hydratase	cytosol	map00630	213.557	60.750	22.837	-1.814 (-)	-1.412 (-)
BGIOSGA006800	Malate dehydrogenase	chloroplast stroma	map00630	10.913	25.110	79.393	1.202 (-)	1.661 (+)
BGIOSGA016887	Malate dehydrogenase	chloroplast stroma	map00630	2.303	6.410	2.433	1.477 (+)	-1.398 (-)
BGIOSGA004108	Malate dehydrogenase	chloroplast stroma	map00630	70.433	167.693	49.720	1.251 (+)	-1.754 (-)
BGIOSGA018498	Ribulose bisphosphate carboxylase small chain	chloroplast membrane	map00630	10536.510	17114.630	37274.950	0.700 (*)	1.123 (+)
BGIOSGA020897	Glycine dehydrogenase (decarboxylating) 2	Mitochondrion	map00630	595.333	185.860	597.616	-1.679 (-)	1.685 (+)
BGIOSGA026137	Peroxisomal	cytoskeleton	map00630	0.627	5.990	1.117	3.256 (+)	-2.423 (-)
BGIOSGA026138	Peroxisomal (S)-2-hydroxy-acid oxidase GLO4	Unkown	map00630	84.913	19.477	7.807	-2.124 (-)	-1.319 (-)
BGIOSGA004733	Malate dehydrogenase	chloroplast	map00630	2.590	7.257	1.347	1.486 (+)	-2.430 (-)

		stroma							
BGIOSGA004212	D-glycerate 3-kinase	chloroplastic	map00630	158.947	67.343	217.867	-1.239 (-)	1.694 (+)
BGIOSGA023784	Ferredoxin-dependent glutamate synthase	chloroplastic	map00630	385.827	138.980	571.433	-1.473 (-)	2.040 (+)
BGIOSGA004313	Glycine dehydrogenase (decarboxylating)	mitochondrial	map00630	113.370	128.810	597.437	0.184 (*)	2.214 (+)
BGIOSGA014826	Phosphoglycolate phosphatase 1A	chloroplastic	map00630	275.037	220.257	865.077	-0.320 (*)	1.974 (+)
BGIOSGA013524	Serine hydroxymethyltransferase	mitochondrial	map00630	580.627	377.427	1262.997	-0.621 (*)	1.743 (+)
BGIOSGA024190	Isocitrate lyase	chloroplast	map00630	7.210	7.383	32.877	0.034 (*)	2.155 (+)
Linoleic acid metabolism								
BGIOSGA007686	Ent-isokaurene C2-hydroxylase	Membrane	map00591	12.133	3.090	5.767	-1.973 (-)	0.900 (*)
BGIOSGA007684	Cytochrome P450 71D8	integral component of membrane	map00591	8.820	2.387	6.630	-1.886 (-)	1.474 (+)
BGIOSGA008468	Ent-isokaurene C2-hydroxylase	Membrane	map00591	23.980	4.070	15.043	-2.559 (-)	1.886 (+)
BGIOSGA018026	Cytochrome P450 71A1	microsome	map00591	3.757	0.170	0.533	-4.466 (-)	1.649 (+)
BGIOSGA006958	Lipoxygenase 2.3	Chloroplast	map00591	28.737	5.963	37.103	-2.269 (-)	2.637 (+)
BGIOSGA036016	Lipoxygenase 2.2	Chloroplast	map00591	16.160	16.923	34.760	0.067 (*)	1.038 (+)
BGIOSGA019579	Linoleate 9S-lipoxygenase 6	Secreted	map00591	13.787	6.057	13.513	-1.187 (-)	1.158 (+)
BGIOSGA013421	Phospholipase A2 homolog 3	Secreted	map00591	12.750	42.657	8.210	1.742 (+)	-2.377 (-)
BGIOSGA007690	Cytochrome P450 71D7	integral component of membrane	map00591	0.240	9.250	38.437	5.268 (+)	2.055 (+)
BGIOSGA000776	Triacylglycerol lipase SDP1	Peroxisome	map00591	7.977	2.150	5.477	-1.892 (-)	1.349 (+)
BGIOSGA028970	lipoxygenase 8	chloroplastic	map00591	1.180	1.113	3.370	-0.084 (*)	1.598 (+)
BGIOSGA037589	Lipoxygenase 2.2	chloroplastic	map00591	27.173	24.960	57.167	-0.123 (*)	1.196 (+)

BGIOSGA013391	Lipoxygenase	chloroplastic	map00591	0.300	0.173	0.523	-0.794 (-)	1.596 (+)
BGIOSGA006215	Ent-cassadiene hydroxylase	Membrane	map00591	1.247	0.663	3.643	-0.911 (*)	2.458 (+)
Phenylpropanoid biosynthesis								
BGIOSGA033360	Peroxidase 5	Secreted	map00940	0.940	15.660	3.197	4.058 (+)	-2.292 (-)
BGIOSGA019136	Peroxidase	Secreted	map00940	0.027	0.877	0.087	5.022 (+)	-3.333 (-)
BGIOSGA014191	Peroxidase 16	Secreted	map00940	0.227	13.840	1.370	5.930 (+)	-3.337 (-)
BGIOSGA013561	Beta-glucosidase BoGH3B	extracellular region	map00940	0.023	1.747	0.173	6.247 (+)	-3.336 (-)
BGIOSGA030124	Cinnamoyl-CoA reductase 1	Cytoplasm	map00940	0.000	1.787	0.063	NO (+)	-4.818 (-)
BGIOSGA029544	Beta-glucosidase 29	Vacuole	map00940	0.847	19.250	2.657	4.507 (+)	-2.857 (-)
BGIOSGA004170	Peroxidase 19	Secreted	map00940	1.057	17.057	1.697	4.013 (+)	-3.330 (-)
BGIOSGA014157	Agmatine hydroxycinnamoyltransferase 1	chloroplast	map00940	0.000	0.460	1.980	NO (+)	2.106 (+)
BGIOSGA021286	Cytochrome P450	Membrane	map00940	0.060	14.050	0.503	7.871 (+)	-4.803 (-)
BGIOSGA017101	Cinnamyl alcohol dehydrogenase 7	plasma membrane	map00940	0.067	2.027	0.020	4.926 (+)	-6.663 (-)
BGIOSGA017307	transferase family protein	Unkown	map00940	0.033	0.090	0.943	1.433 (+)	3.390 (+)
BGIOSGA006537	3-aminomethylindole N-methyltransferase	Unkown	map00940	31.707	1.657	18.963	-4.258 (-)	3.517 (+)
BGIOSGA011559	Peroxidase	Secreted	map00940	0.133	0.893	0.063	2.744 (+)	-3.818 (-)
BGIOSGA012978	Peroxidase	Secreted	map00940	0.050	0.727	0.043	3.861 (+)	-4.068 (-)
BGIOSGA014724	beta-glucosidase 15	Endoplasmic reticulum lumen	map00940	0.137	0.253	2.957	0.890 (*)	3.545 (+)
BGIOSGA036496	Peroxidase 43	Secreted	map00940	4.387	32.477	2.857	2.888 (+)	-3.507 (-)
BGIOSGA031327	4-coumarate--CoA ligase-like 2	Peroxisome	map00940	1.080	0.363	7.093	-1.572 (-)	4.287 (+)

Photosynthesis - antenna proteins								
BGIOSGA014978	Chlorophyll a-b binding protein CP24	chloroplast thylakoid membrane	map00196	784.490	1699.733	3049.067	1.115 (+)	0.843 (*)
BGIOSGA022800	Chlorophyll a-b binding protein 1B-21	Chloroplast	map00196	4999.083	2877.400	6113.020	-0.797 (*)	1.087 (+)
BGIOSGA030468	Chlorophyll a-b binding protein 7	Chloroplast	map00196	18.323	16.500	38.620	-0.151 (*)	1.227 (+)
BGIOSGA026928	Chlorophyll a-b binding protein P4	Chloroplast	map00196	3380.987	2042.950	4857.027	-0.727 (*)	1.249 (+)
BGIOSGA025944	Chlorophyll a-b binding protein of LHCII type III	Chloroplast	map00196	1458.830	2094.607	4577.870	0.522 (*)	1.128 (+)
BGIOSGA035064	Chlorophyll a-b binding protein CP26	Chloroplast	map00196	1660.937	2693.557	5665.997	0.698 (*)	1.073 (+)
BGIOSGA024096	Chlorophyll a-b binding protein CP29.1	Chloroplast	map00196	2988.090	3004.310	6380.403	0.008 (*)	1.087 (+)
BGIOSGA003922	Chlorophyll a-b binding protein 2	Chloroplast	map00196	4230.810	5860.343	12399.560	0.470 (*)	1.081 (+)
BGIOSGA026930	Chlorophyll a-b binding protein P4	chloroplastic	map00196	1.853	1.170	3.357	-0.664 (*)	1.521 (+)
BGIOSGA026003	Chlorophyll a-b binding protein 7	chloroplastic	map00196	1752.437	1366.087	2935.717	-0.359 (*)	1.104 (+)
BGIOSGA006945	Photosystem I chlorophyll a/b-binding protein 3-1	chloroplastic	map00196	2316.160	1600.787	4143.507	-0.533 (*)	1.372 (+)
Ribosome								
BGIOSGA027921	60S ribosomal protein L37-1	Cytosol	map03010	41.117	81.240	27.810	0.982 (*)	-1.547 (-)
BGIOSGA031729	60S ribosomal protein L23	Cytosol	map03010	421.067	877.967	275.930	1.060 (+)	-1.670 (-)
BGIOSGA031035	60S ribosomal protein L32-1	Cytosol	map03010	99.800	230.367	56.933	1.207 (+)	-2.017 (-)
BGIOSGA034879	Syntaxin-61	Golgi apparatus	map03010	5.307	19.047	5.077	1.844 (+)	-1.908 (-)

BGIOSGA026715	60S ribosomal protein L31	Cytosol	map03010	62.920	188.727	45.177	1.585 (+)	-2.063 (-)
BGIOSGA009558	40S ribosomal protein S23	Cytosol	map03010	12.447	41.747	0.987	1.746 (+)	-5.403 (-)
BGIOSGA003154	50S ribosomal protein L3-2	Cytosol	map03010	19.427	4.640	1.020	-2.066 (-)	-2.186 (-)
BGIOSGA007303	60S ribosomal protein L9	Cytosol	map03010	85.847	123.920	24.720	0.530 (*)	-2.326 (-)
BGIOSGA031945	RP-L32	ribosome	map03010	8.500	5.650	1.470	-0.589 (*)	-1.942 (-)
BGIOSGA031297	Ubiquitin-60S ribosomal protein L40-2	Cytoplasm and Cytosol	map03010	102.253	127.107	25.217	0.314 (*)	-2.334 (-)
BGIOSGA000060	40S ribosomal protein S10-2	Cytoplasm and Cytosol	map03010	38.710	56.873	10.047	0.555 (*)	-2.501 (-)
BGIOSGA001297	50S ribosomal protein L25	Cytoplasm and Cytosol	map03010	11.723	4.663	0.733	-1.330 (-)	-2.669 (-)
BGIOSGA025984	40S ribosomal protein S13-2	Cytoplasm and Cytosol	map03010	23.217	19.510	3.723	-0.251 (*)	-2.390 (-)
Ribosome biogenesis in eukaryotes								
BGIOSGA010733	nucleolar protein 5-2	nucleolus	map03008	50.243	10.630	2.920	-2.241 (-)	-1.864 (-)
BGIOSGA023359	WD repeat-containing protein 36	Cytoskeleton	map03008	13.657	2.713	0.887	-2.331 (-)	-1.614 (-)
BGIOSGA037067	RNA cytidine acetyltransferase 1	Nucleus	map03008	14.190	2.347	0.873	-2.596 (-)	-1.426 (-)
BGIOSGA007301	U3 small nucleolar RNA-associated protein 6 homolog	Nucleus	map03008	12.863	2.303	0.950	-2.481 (-)	-1.278 (-)
BGIOSGA012435	WD repeat-containing protein 43	Nucleus	map03008	16.643	3.537	1.110	-2.234 (-)	-1.672 (-)
BGIOSGA035016	Calmodulin-interacting protein 111	Chloroplast	map03008	3.507	0.573	0.110	-2.613 (-)	-2.382 (-)
BGIOSGA027776	nucleolar protein 5-2	Nucleus	map03008	7.247	1.127	3.397	-2.685 (-)	1.592 (+)
BGIOSGA029069	Nucleolar protein 6	Nucleus	map03008	1.160	4.147	1.033	1.838 (+)	-2.005 (-)
BGIOSGA037054	Nucleolar protein 6 isoform X2	Nucleus	map03008	12.950	1.947	0.560	-2.734 (-)	-1.798 (-)
BGIOSGA031778	Midasin	Nucleus	map03008	10.700	2.313	0.750	-2.210 (-)	-1.625 (-)

BGIOSGA022681	H/ACA ribonucleoprotein complex subunit 2-like protein	Nucleus	map03008	32.123	12.583	1.750	-1.352 (-)	-2.846 (-)
BGIOSGA009310	Mediator of RNA polymerase II transcription subunit 36a	Nucleus	map03008	74.960	36.933	9.447	-1.021 (-)	-1.967 (-)
BGIOSGA012722	H/ACA ribonucleoprotein complex subunit 4	Nucleus	map03008	7.540	2.263	0.257	-1.736 (-)	-3.140 (-)
BGIOSGA012720	Uncharacterized protein	Nucleus	map03008	1.880	0.580	0.047	-1.697 (-)	-3.636 (-)
BGIOSGA025408	Protein SLOW WALKER 1	Nucleus	map03008	16.330	8.243	1.897	-0.986 (-)	-2.120 (-)
BGIOSGA023851	H/ACA ribonucleoprotein complex subunit 4	Nucleus	map03008	23.400	6.720	0.600	-1.800 (-)	-3.485 (-)
BGIOSGA035498	H/ACA ribonucleoprotein complex subunit 1-like protein 1	Nucleus	map03008	45.980	25.733	3.283	-0.837 (*)	-2.970 (-)
BGIOSGA010727	Nucleolar protein 56	Nucleus	map03008	50.013	16.233	3.343	-1.623 (-)	-2.280 (-)
Starch and sucrose metabolism								
BGIOSGA013561	Beta-glucosidase BoGH3B	extracellular	map00500	0.023	1.747	0.173	6.226 (+)	-3.333 (-)
BGIOSGA022241	Granule-bound starch synthase 1, chloroplastic/amyloplastic	Chloroplast	map00500	0.720	0.030	1.823	-4.585 (-)	5.925 (+)
BGIOSGA028837	Alpha-amylase isozyme	Extracellular	map00500	0.043	2.757	0.673	5.991 (+)	-2.034 (-)
BGIOSGA028835	Alpha-amylase isozyme	Extracellular	map00500	0.317	4.317	1.127	3.769 (+)	-1.938 (-)
BGIOSGA020689	alpha-glucosidase	Endoplasmic reticulum	map00500	0.457	5.063	1.430	3.471 (+)	-1.824 (-)
BGIOSGA029544	Beta-glucosidase 29	Extracellular	map00500	0.847	19.250	2.657	4.507 (+)	-2.857 (-)
BGIOSGA014722	Beta-glucosidase 18	Extracellular	map00500	1.577	9.223	3.067	2.548 (+)	-1.589 (-)
BGIOSGA011829	Beta-amylase 2	Chloroplast	map00500	15.397	99.840	195.417	2.697 (+)	0.969 (*)
BGIOSGA023458	alpha-glucosidase	Endoplasmic reticulum	map00500	1.717	10.967	3.877	2.675 (+)	-1.500 (-)

BGIOSGA016364	Beta-fructofuranosidase	Cell wall	map00500	0.223	11.287	2.043	5.659 (+)	-2.466 (-)
BGIOSGA000239	sucrose-phosphate synthase 1	Plasma Membrane	map00500	4.517	6.923	36.113	0.616 (*)	2.383 (+)
BGIOSGA014724	beta-glucosidase 15	Extracellular	map00500	0.137	0.253	2.957	0.890 (*)	3.545 (+)
BGIOSGA028759	alpha,alpha-trehalose-phosphate synthase [UDP-forming] 9	Vacuole	map00500	11.403	5.357	30.943	-1.090 (-)	2.530 (+)
BGIOSGA019723	Beta-glucosidase 22	Endoplasmic reticulum	map00500	103.717	18.417	244.370	-2.494 (-)	3.730 (+)
BGIOSGA012138	Glucan endo-1,3-beta-glucosidase 3	Plasma membrane	map00500	0.303	1.197	0.050	1.980 (+)	-4.581 (-)
BGIOSGA024424	Granule-bound starch synthase 1b	chloroplastic	map00500	12.697	8.970	68.577	-0.501 (*)	2.935 (+)
BGIOSGA033092	Beta-amylase 1	chloroplastic	map00500	1.017	1.713	8.830	0.753 (*)	2.366 (+)

Note: "+": Upregulated

"-": Downregulated

"*": No significance

"NO": The genes were not quantified under chilling or subsequent recovery.

Table S5: Primers used for qRT-PCR

Gene	Forward sequence（5’-3’）	Reverse sequence（5’-3’）
lipoxygenase 2.2	CTTTAGCTCGAGTACAGAGAGG	CGCCGAAGCTAGACTCATATAT
lipoxygenase	CTTTAGCTCGAGTACAGAGGAC	TACGAAAGTTGGATGCTGAGAG
60S ribosomal protein L23A	GAAGTCTGGGTCAATCAAGAGA	CAAGGGTGTTGTTGTCTTCAAT
60S ribosomal protein L32	GAAAGTTCAAGGGATGCACTTT	AATTTGTTGGGAAGGTAATGCC
40S ribosomal protein S23	CCGACAAAGCATACAAGAAGAG	AATCTTTTCAAGGACAATGCCC
60S ribosomal protein L31	CAATGCCATCAAAGAGATCAGG	TTAATCCGTTTCGTCAACAACC
Actin	TGCTATGTACGTCGCCATCCAG	AATGAGTAACCACGCTCCGC

Fig. S1 The Venn diagram analysis of three different temperature treatments. TR vs CT: The low temperature stress compared with normal temperature treatment in Dular. RC vs TR: The return to normal temperature treatment compared with low temperature stress in Dular.

Fig. S2 The different genes expression in Dular under three different temperature treatments. CT: Normal-temperature treatment. TR: Low-temperature treatment. RC: Recovery to normal-temperature treatment.

Fig. S3 Heatmap analysis of the genes under three different temperature treatment. CT: Normal-temperature treatment. TR: Chilling treatment. RC: Recovery to normal-temperature treatment.

Fig. S1

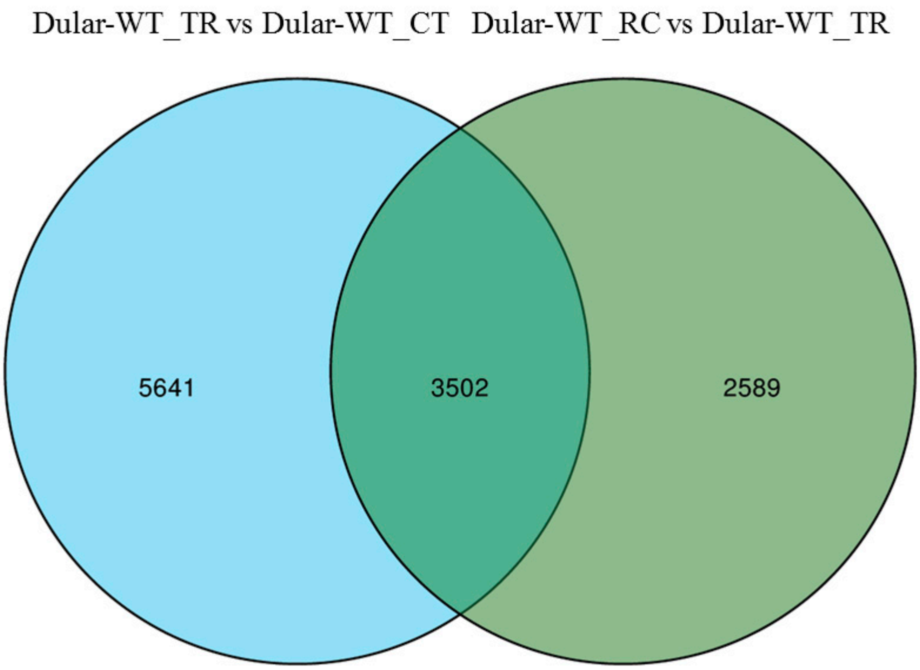

Fig. S2

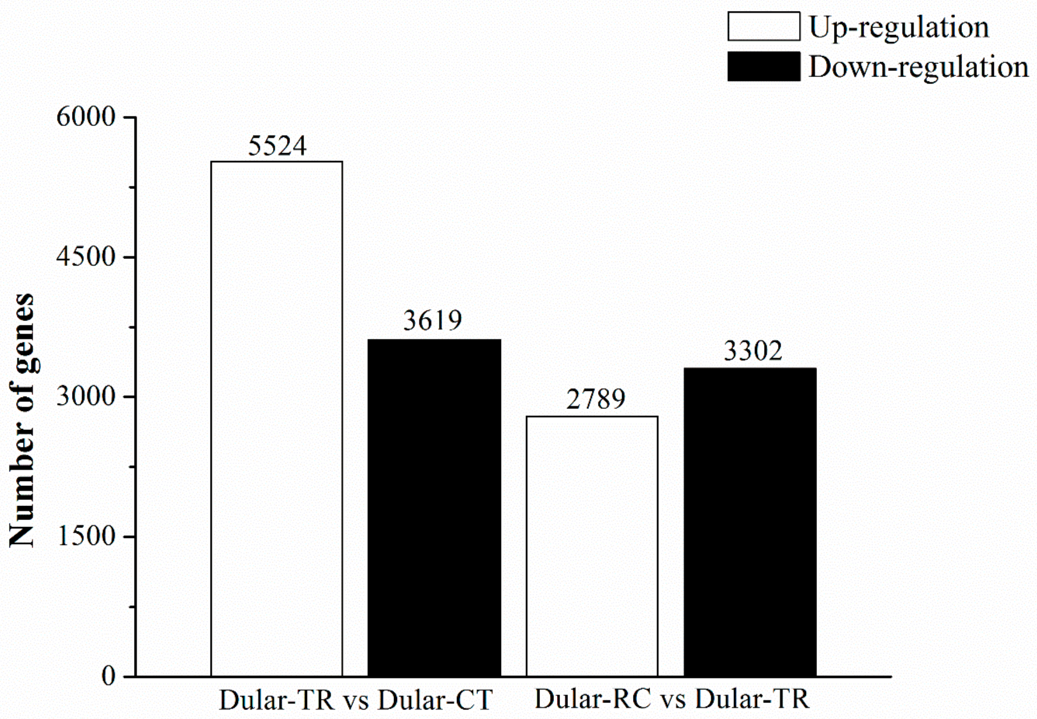
Up-regulation
Down-regulation
6000
4500
3000
1500
0
Number of genes
5524
3619
2789
3302
Dular-TR vs Dular-CT
Dular-RC vs Dular-TR

Fig. S3

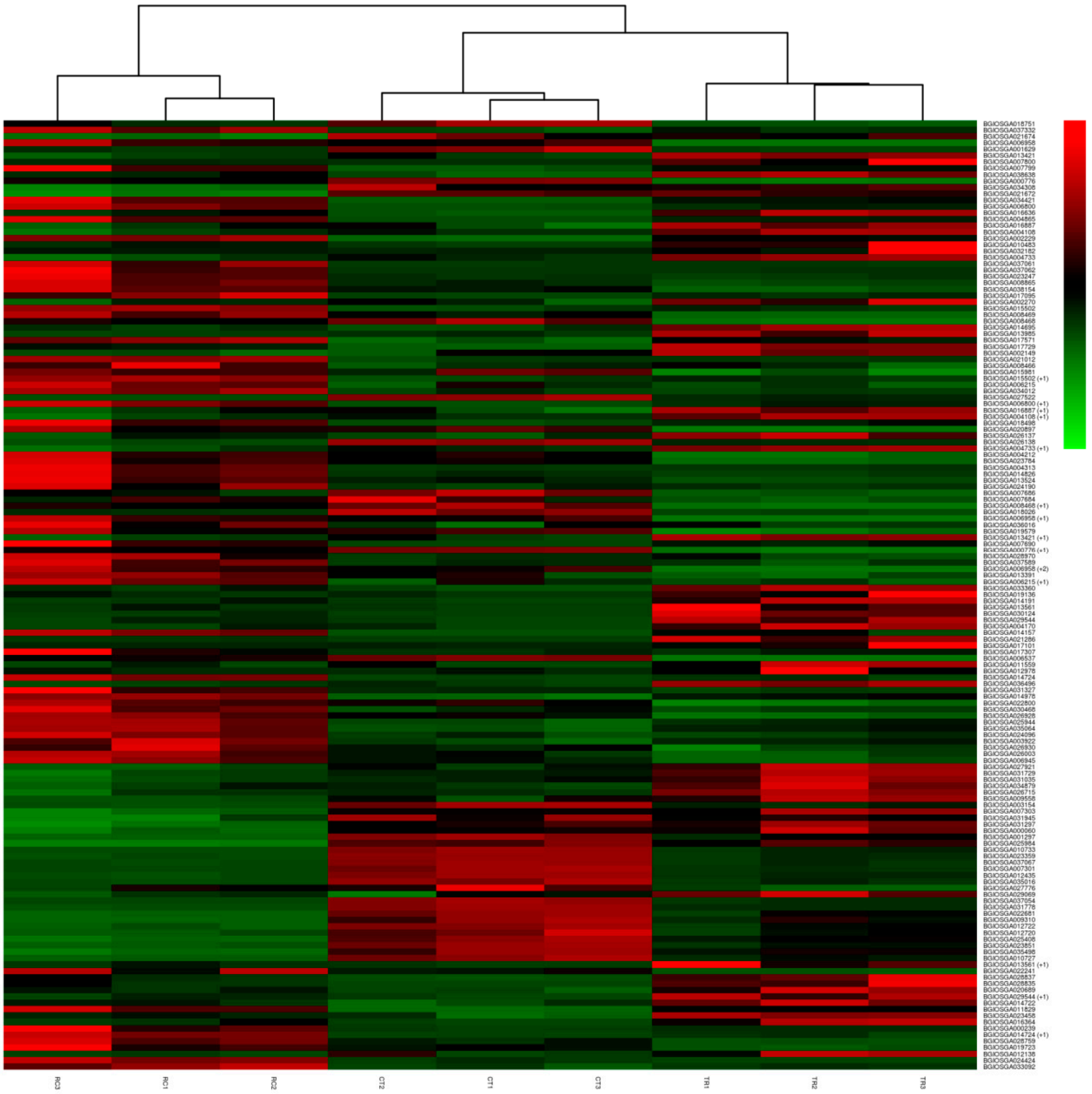

Supplement: Supplementary file 1 [file ijms-23-10739-s001.zip › ijms-1905282-Supplementary material.pdf]
