# Supplementary figures and images for: Transcriptome Analysis of the Responses of Rice Leaves to Chilling and Subsequent Recovery
Source: Int J Mol Sci. 2022 Sep 15;23(18):10739. doi: 10.3390/ijms231810739 (PMC9502032; doi:10.3390/ijms231810739)

# Histogram of KEGG(RC\_TR\_20220417)

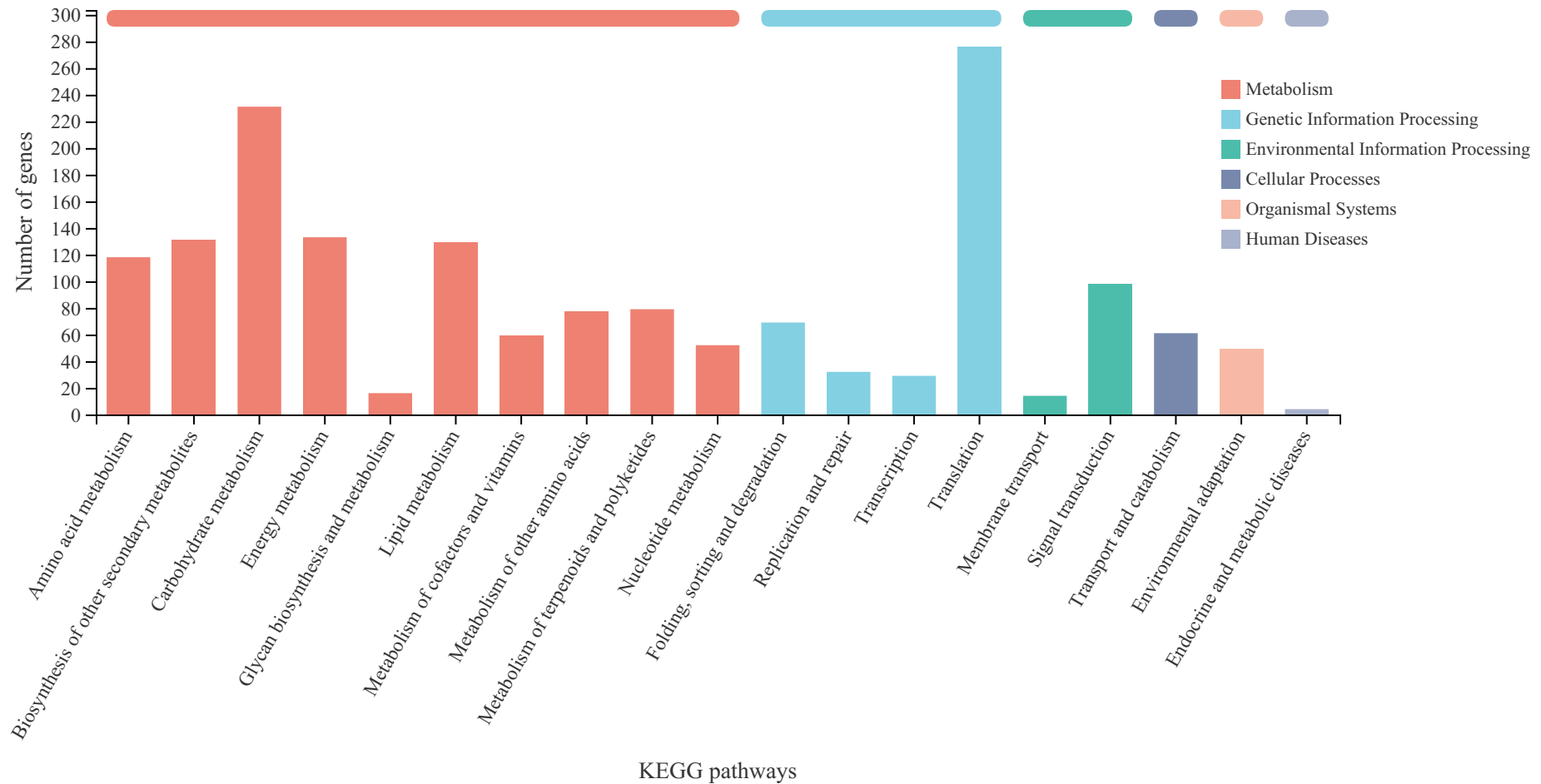

Supplement: Supplementary file 1 [file ijms-23-10739-s001.zip › Histogram of Pathway classification statistics in RC_TR.pdf]

# Histogram of KEGG(TR\_CT)

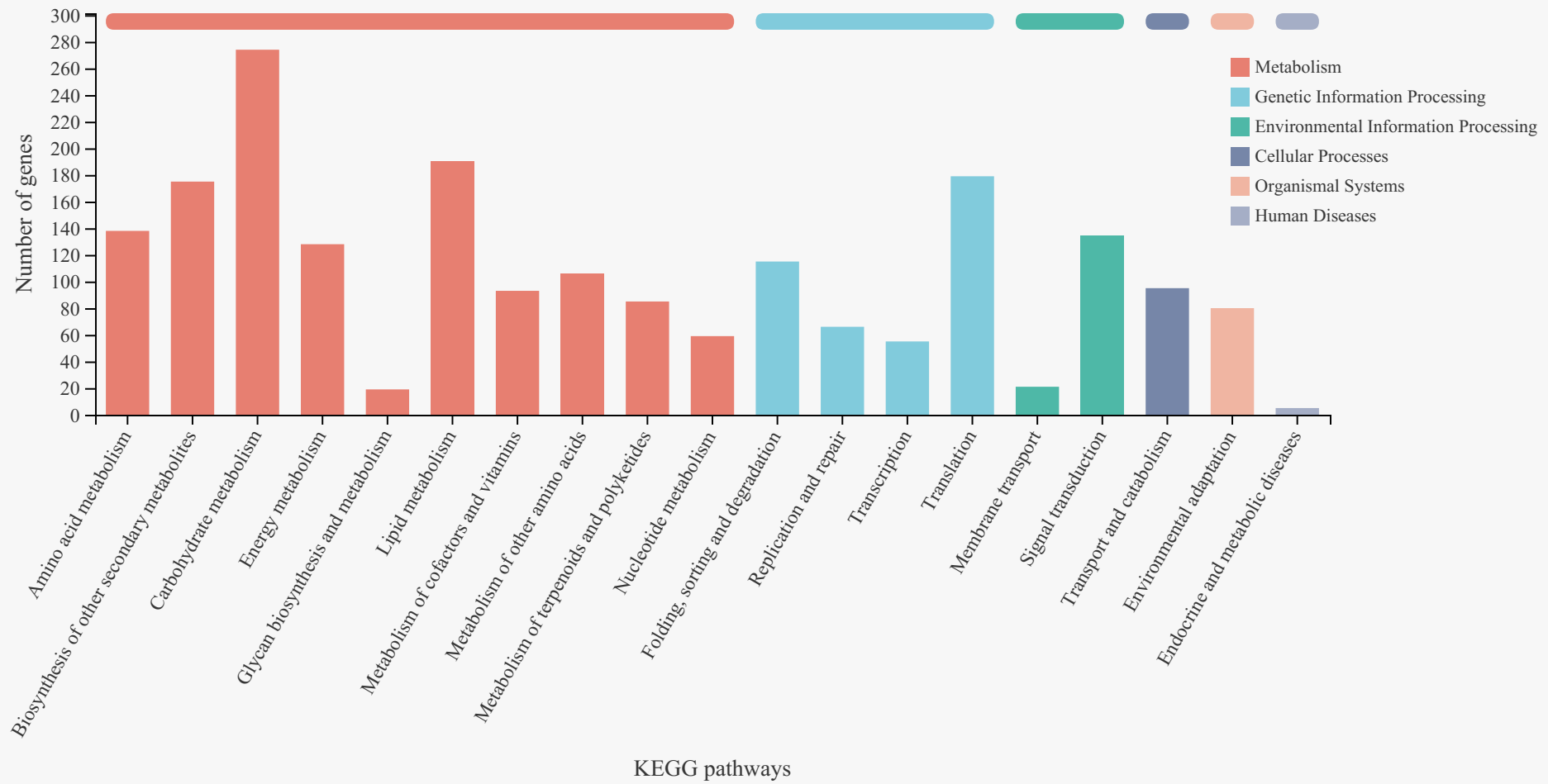

Supplement: Supplementary file 1 [file ijms-23-10739-s001.zip › Histogram of Pathway classification statistics in TR_CT.pdf]
